# Normalisation of mental health problems: Adolescents’ views on mental health problems and stigma

**DOI:** 10.1093/eurpub/ckac130.206

**Published:** 2022-10-25

**Authors:** V Hermann, N Durbeej, A-C Karlsson, A Sarkadi

**Affiliations:** Department of Public Health and Caring Sciences, Uppsala University, Uppsala, Sweden

## Abstract

**Background:**

It is essential to listen to adolescents’ views on mental health issues since these problems are common among young people. Exposure to stigmatization is an additional burden, causing increased suffering. This qualitative descriptive study aimed to explore adolescents’ views on the prevalence of mental health problems and public stigma related to mental health problems.

**Methods:**

Semi-structured individual interviews and focus group interviews were conducted with a total of 32 adolescents, aged 15-18 years. The interviews were held on Gotland, Sweden's largest island, between October and December 2020. Systematic text condensation was used to analyse the data.

**Results:**

Three themes were identified: Having mental health problems is the new normal; What others think of us affects us a lot; If others lack experience and knowledge, they don't respond in a good way. The adolescents perceived mental health problems as a common phenomenon. Increased mental health problems in young people were linked to pressure related to school performance, social media and improved openness about mental health problems. Stereotypic gender norms, rumours and prejudice were perceived as important causal risk factors of mental health problems. Lack of knowledge was suggested as a source of prejudice against people suffering from mental health problems.

**Conclusions:**

The adolescents recognised mental health problems as an increasing public health issue, but also as a normal phenomenon due to current living conditions for young people.. They perceived stereotypic gender norms, taboo and prejudice against mental health problems as factors contributing to and increasing mental health problems and wished for a society without such factors. The results suggest that the tri-folded description of stigma involving stereotypes, prejudice and discrimination can be applicable to adolescents.

**Key messages:**

- The results suggest that adolescents recognise mental health problems as an increasing public health issue but also as a normal phenomenon due to current living conditions for young people.
- Increased knowledge of mental health problems alongside reduction of prejudice and stereotypic gender norms was requested by the adolescents to improve young peoples’ mental health.

